# Cognitive, Emotive, and Cognitive-Behavioral Correlates of Suicidal Ideation among Chinese Adolescents in Hong Kong

**DOI:** 10.1100/tsw.2010.42

**Published:** 2010-03-05

**Authors:** Sylvia Y.C.L. Kwok, Daniel T. L. Shek

**Affiliations:** ^1^ Department of Applied Social Studies, The City University of Hong Kong, Hong Kong; ^2^ Department of Applied Social Sciences, The Hong Kong Polytechnic University, Hong Kong; ^3^ Public Policy Research Institute, The Hong Kong Polytechnic University, Hong Kong; ^4^ Kiang Wu Nursing College of Macau, Macao

**Keywords:** hopelessness, emotional competence, social problem solving, suicidal ideation, Chinese adolescents

## Abstract

Utilizing Daniel Goleman's theory of emotional competence, Beck's cognitive theory, and Rudd's cognitive-behavioral theory of suicidality, the relationships between hopelessness (cognitive component), social problem solving (cognitive-behavioral component), emotional competence (emotive component), and adolescent suicidal ideation were examined. Based on the responses of 5,557 Secondary 1 to Secondary 4 students from 42 secondary schools in Hong Kong, results showed that suicidal ideation was positively related to adolescent hopelessness, but negatively related to emotional competence and social problem solving. While standard regression analyses showed that all the above variables were significant predictors of suicidal ideation, hierarchical regression analyses showed that hopelessness was the most important predictor of suicidal ideation, followed by social problem solving and emotional competence. Further regression analyses found that all four subscales of emotional competence, i.e., empathy, social skills, self-management of emotions, and utilization of emotions, were important predictors of male adolescent suicidal ideation. However, the subscale of social skills was not a significant predictor of female adolescent suicidal ideation. Standard regression analysis also revealed that all three subscales of social problem solving, i.e., negative problem orientation, rational problem solving, and impulsiveness/carelessness style, were important predictors of suicidal ideation. Theoretical and practice implications of the findings are discussed.

### INTRODUCTION

There are different cognitive, emotive, and cognitive-behavioral theories that explain suicidal behaviors. First, Aaron Beck's concept of the "cognitive triad", i.e., negative thoughts about oneself, the future, and others, was central to the cognitive theory of suicide[1]. Beck's cognitive perspective also states that hopelessness is the most important clinical variable implicated in suicidal behavior. Empirically, a number of studies also found that there was a significant relationship between hopelessness and suicidality[e.g., 2]. In a longitudinal study of Hong Kong Chinese and Caucasian American adolescents[3], hopelessness was found to be the strongest of cognitive variables in concurrent association with suicidal ideation in bivariate and multivariate models[4]. However, some studies found that depression was a more powerful predictor of suicidal ideation than was hopelessness[5]. Since depression is a chronic illness and requires long-term treatment, this study will focus on examining the significance of hopelessness as a cognitive factor in suicidal ideation for its practical value, with reference to Beck's cognitive theory.

According to Daniel Goleman's theory of emotional competence[6], a person with high emotional intelligence has better emotional self-awareness and higher empathy, and is more competent in managing his/her own emotions and handling interpersonal relationships; hence, that person will have better psychological well-being. Empirical studies also stated that there was an emotional element in suicidal ideation. A number of studies have indicated the importance of emotional expression and emotion regulation[e.g., 7] in working with acutely suicidal individuals. Previous research also suggested that depressed adolescents had difficulty regulating negative affect, and had fewer and less-effective strategies for emotion regulation[8]. In addition, four emotional competencies were found to be important in understanding how well people deal with stress and avoid poor mental health outcomes: perceiving emotions, managing self-relevant emotions, managing others' emotions, and intention to seek help when in emotional need.

However, one study found that at some high extremes of temperament, emotion regulation had no impact on suicidality[7]. In addition, another study revealed that there were gender differences in the relationship between emotion regulation and depressive symptoms[9]. Women with high depressive symptoms were found to exhibit greater attention to emotions, more impaired antirumination emotional repair strategies, and greater reports of depressive symptoms than men with high depressive symptoms. A study of Chinese youth found that the girls were significantly higher than boys in terms of effortful control, affability, and negative mood, but lower on surgency and aggression[10]. Research also showed that females express emotion more than males, due to maternal behavior[11]. Since the findings are equivocal and there are differences in emotional expression for the Chinese males and females, the relationship between emotional competence and adolescent suicidal ideation, with reference to gender differences, is worth further examination.

D'Zurilla and Maydeu-Olivares made a distinction between two types of problem solving measures: process measures and outcome measures. As a process measure, social problem solving is a cognitive-behavioral construct that includes problem orientation and problem solving proper[12,13]. Theoretically, Rudd[14] also suggested a cognitive-behavioral theory of suicidality, elaborating on Beck's modal theory of psychopathology. According to Rudd, suicidality was associated with the behavioral system, cognitive system, physiological system, and affective system, while social problem solving was one of the cognitive-behavioral constructs. Empirically, studies indicated that social problem solving was closely linked with suicidal behavior[e.g., 15]. However, the literature on the relationship between social problem solving and adolescent suicide is not conclusive. On the one hand, there are research findings showing that suicide attempters showed deficits in problem orientation and had difficulty with the generation of alternatives, decision making, and solution implementation and verification[16]. On the other hand, there are studies showing no significant problem-solving differences between groups of suicidal psychiatric inpatients, nonsuicidal psychiatric inpatients, and community high school students[e.g., 17]. In addition, a study found that female adolescents' lower levels of positive thinking, higher scores on negative problem orientation, and self-focused negative cognitions partially mediated gender differences in depressive symptoms[18]. A study with Hong Kong Chinese students showed that girls tended to cope with failure by seeking social support[19]. Another study with Hong Kong secondary school students found that boys had significantly higher scores in rational problem solving and impulsiveness/carelessness style than girls[20]. As there are differences in the problem-solving strategies for the Chinese males and females, the relationship between social problem solving and adolescent suicidal ideation, with reference to gender differences, needs to be further explored.

Theoretically, emotional competence theory, cognitive theory, and cognitive-behavioral theory can be used to examine suicidal ideation. Empirically, although Williams and Pollock[21] highlighted emotional regulation, hopelessness, and interpersonal problem solving as mediating factors in suicidal behavior, there are controversial results in the studies on the relative importance of hopelessness, social problem solving, and emotional competence in predicting suicidal ideation. Results in a study examining the relationship between social and emotional competence and mental health[22] revealed that all social and emotional competence measures, including social problem-solving skills, effective emotional control, and level of emotional awareness, had significant incremental value over stressful events in predicting social and mental health. A study showed that both problem solving and emotional discharge, as coping strategies, were negatively related with suicidal behavior[23]. Although hopelessness and problem-solving deficits were highlighted as psychological processes relevant to the understanding of suicidality, a study showed that problem-solving confidence predicted depressive symptoms and hopelessness, but not suicidal ideation in Asian international students[24]. Some other studies found that even after hopelessness was statistically controlled, emotion-oriented coping remained a significant predictor of suicidal ideation in Western people[e.g., 25]. Since the results are equivocal, it is interesting to examine the relative importance of the emotional, cognitive, and cognitive-behavioral factors in predicting suicidal ideation. Previous studies stated that there was significant association between exposure to single parenthood and suicidal behavior[e.g., 26]. Research also highlighted that parents' educational level was associated with adolescents' mental health[27]. Hence, it is worthwhile to examine whether the same association was found among Chinese adolescents.

However, there are several limitations intrinsic to the previous studies. First, the studies on the relationship among different measures of cognitive, emotive, and cognitive-behavioral correlates, and adolescent suicidal ideation, are inconclusive. Second, few studies have included the cognitive, emotive, and cognitive-behavioral components in a single study. However, it can be argued that it is both theoretically and empirically important to include all three correlates in a single study, and to assess their relative contribution to adolescent suicidal ideation.

Third, the sample size in the existing studies was either too small or was limited to a homogenous subsample. Fourth, most of the measuring instruments for the studies conducted in Hong Kong were adapted from the West and may not be able to capture the essence of the Chinese culture. Fifth, there is a predominance of Western studies regarding the relationship between the cognitive, emotive, and cognitive-behavioral factors, and adolescent suicidal ideation. A survey of the PsycINFO in January 2010 with the search term of "suicidal ideation" showed that while there were 5,323 citations, there were only 63 citations when the search terms of "suicidal ideation" and "Chinese" were used. As Chinese people constitute roughly one-fifth of the world's population, our study is an important contribution to fill the gap.

Against the above background, several research hypotheses were addressed in this study. (1) What is the relationship between hopelessness, emotional competence, social problem solving, and Chinese adolescent suicidal ideation in Hong Kong? Based on the theoretical frameworks of cognitive theory, emotional competence theory, and cognitive-behavioral theory, as well as previous research findings, it was hypothesized that (a) hopelessness would have a positive relationship with suicidal ideation (Hypothesis 1), (b) emotional competence would have a negative relationship with suicidal ideation (Hypothesis 2), and (c) social problem solving would have a negative relationship with suicidal ideation (Hypothesis 3). (2) What is the relative importance of the above correlates in predicting Chinese adolescent suicidal ideation in Hong Kong? Since previous studies highlighted the significant role of hopelessness in predicting suicidal behavior[4,28], it was hypothesized that hopelessness would be the most important predictor of suicidal ideation. However, as Chinese people may not place too much emphasis on emotional expression when compared with people from the West, emotion may occupy a relatively less-important position[29]. Hence, it was hypothesized that emotional competence would be the least important predictor of suicidal ideation (Hypothesis 4).

## METHODS

### Participants

A cross-sectional survey was conducted and secondary schools were approached by convenience sampling with the help of agencies providing school social work service. Of the 68 schools (out of a total of 426 secondary schools in Hong Kong) that were contacted, 42 schools (among these self-financed, subvented, and government schools) from Hong Kong Island, Kowloon, and the New Territories agreed to join the study. The response rate was 62%, which is comparable with a similar study on suicidal ideation and behavior[22]. By the end of the survey period, a total of 5,557 valid questionnaires were collected. With reference to the total secondary student population in Hong Kong at the time the study was conducted (328,573 students), the sample size used (2%) can be regarded as adequate. There were slightly more males (53.1%) than females (46.9%) in the sample, which reflects the distribution among the general population. The participants were evenly distributed among different forms (25.8% in Secondary 1, 24.7% in Secondary 2, 24.4% in Secondary 3, 25.1% in Secondary 4). The age of the respondents varied from 11 to 18 years, with an overall mean of 13.87 years ($SD$ = 1.47). A large proportion (81.0%) of the participants reported their fathers to have a full-time job, while 10.0% had a part-time job, and 9.0% were homemakers or unemployed. A small proportion (23.7%) of the fathers had primary or no education, while 66.4% received secondary education, and 9.9% received tertiary education or above. Meanwhile, 42.3% of the mothers had a full-time job, while 15.6% had a part-time job, and 42.0% were homemakers or unemployed. About one-quarter (26.1%) of the mothers had primary or no education, while 67.4% received secondary education, and 6.4% received tertiary education or above. A large proportion (87.7%) of the families of the participants was reported to be intact and 12.3% reported having nonintact families (i.e., single-parent families due to death of a parent, divorce, or separation). About one-third (35.6%) of the participants had a family monthly income lower than HK$10,000 (about US$1,282). A small proportion (13.1%) of the participants' families received Comprehensive Social Security Assistance (CSSA).

### Procedures

School, parental, and participant consent were obtained prior to data collection. During the survey, a school social worker, a teacher, or a research assistant was present to give a short briefing on the general aims and the confidentiality of the study, and they were present throughout the whole administration process to answer queries raised by the participants. Briefing the participants and filling out the questionnaires took around 40 min.

### Instruments

- **Hopelessness Subscale of the Chinese Hopelessness Scale (C-HOPE)** — The original English scale was devised by Beck et al.[30], and then translated to Chinese and further developed by Shek[31]. The subscale has 10 items and the respondents were asked to choose an answer from a 4-point Likert scale ranging from "strongly agree" to "strongly disagree", with a minimum total score of 10 and a maximum total score of 40. A higher score indicates a higher degree of hopelessness. According to Shek and Lee's study[32], the scale possessed adequate construct validity. The scale was demonstrated to be internally consistent in this study ($\alpha$ = 0.88).
- **Short Form of the Chinese Emotional Intelligence Scale (C-EIS-R)** — Developed by Chan[33], the instrument was adapted from the English 33-item EIS originally developed by Schutte and colleagues[34]. The short form of the 12-item C-EIS-R has four empirical subscales to represent four dimensions of emotional intelligence: self-management of emotions, social skills, empathy, and utilization of emotions. Respondents indicate their agreement to each of the 12 statements using a 5-point Likert scale ranging from “strongly agree” to “strongly disagree”, with a minimum total score of 12 and a maximum total score of 60. A higher score indicates better emotional competence. The scale was found to be internally consistent in this study ($\alpha$ = 0.79).

- **Short Form of the Chinese Version of the Social Problem Solving Inventory (C-SPSI-R)** — Developed by Siu and Shek[35], the instrument was adapted from the 52-item Social Problem-Solving Inventory Revised (SPSI-R)[11]. A validation study was conducted locally with 352 secondary school students[35]. The short form of the 25-item C-SPSI-R had a five-factor structure that was largely consistent with the original English SPSI-R. However, the three-factor model of the C-SPSI[35] was used in this study because the subscales had adequate coverage on the social problem-solving concept and the internal consistency of the three subscales was good. The three subscales are negative problem orientation (NPO), rational problem solving (RPS), impulsiveness/carelessness style (ICS). For each of the 25 items, the respondents are requested to choose an answer on a 5-point Likert scale ranging from “not at all true of me” to “extremely true of me”. In calculating the total score of the C-SPSI-R, the items in the subscales of NPO and ICS were reversed, resulting in a minimum total score of 0 and a maximum total score of 100. Half of the test items indicate a constructive orientation toward problem solving, while the other half indicates a dysfunctional or inhibitive orientation. The items are presented in a random order. A higher score indicates better social competence. There were findings showing that the related measure were valid and reliable[35]. Reliability analyses showed that this abridged version of C-SPSI-R was reliable in this study ($\alpha$ = 0.83).
- **Suicidal Ideation Subscale (C-SIS) of the Suicidal Risk Scale** — The scale was locally developed by Tse and Bagley[36]. For each of the 13 items, respondents were required to choose an answer from a 4-point Likert scale ranging from “strongly agree” to “strongly disagree”, with a minimum total score of 13 and a maximum total score of 52. A higher score indicates a higher level of suicidal ideation. A previous validation study[36] showed that the C-SIS has high reliability and validity. Reliability analyses showed that the scale had high reliability in this study ($\alpha$ = 0.93).

### Statistical Analyses

The means, standard deviations, and Cronbach’s alpha ($\alpha$) were first computed. Then, Pearson correlation analyses between the independent variables of the subscales of emotional competence, social problem solving, hopelessness, and the dependent variable of suicidal ideation were carried out, using Bonferroni-corrected alpha level to adjust for multiple comparisons. To examine the relative importance within the subscales of emotional competence and social problem solving in predicting suicidal ideation between the two genders, regression analyses were carried out separately for both genders and the total sample. Lastly, to examine the relative importance of emotional competence, social problem solving, and hopelessness in predicting suicidal ideation, hierarchical regression analyses were performed. For the first step, sociodemographic variables (including participants’ age, fathers’ and mothers’ jobs, fathers’ and mothers’ education, parents’ marital status, monthly family income, CSSA recipients) were entered as control. For the second step, emotional competence and social problem solving were entered into the equation to determine whether they added significant incremental validity to the prediction model. For the final step, hopelessness was entered into the equation to determine its effects on emotional competence and social problem solving. Linear Mixed Model analysis was also performed to examine whether there was any school or banding effects.

### RESULTS

The mean total scores for the Chinese Hopelessness Scale, Chinese Emotional Intelligence Scale, Social Problem Solving Inventory, and Suicidal Ideation Subscale were 20.28 (*SD* = 5.96), 43.95 (*SD* = 6.04), 48.29 (*SD* = 11.51), 22.54 (*SD* = 8.64), respectively (Table 1). Using Pearson correlation analysis, results showed that there was significant positive correlation between adolescent suicidal ideation and hopelessness ($r = 0.68$, $p < 0.001$), hence supporting Hypothesis 1. Adolescent suicidal ideation increased when hopelessness increased. Standard regression analysis showed that hopelessness was a significant predictor of adolescent suicidal ideation ($R^2 = 0.47$).

**TABLE 1**
**Descriptive Statistics of Adolescent Suicidal Ideation, Emotional Competence, Social Problem Solving, the Subscales, and Hopelessness in Male, Female, and Total Sample**

| | Male | | Female | | Total | | α |
|---|---|---|---|---|---|---|---|
| | *M* | *SD* | *M* | *SD* | *M* | *SD* | |
| Adolescent suicidal ideation | 22.49 | 8.63 | 22.59 | 8.64 | 22.54 | 8.64 | 0.93 |
| Emotional competence | 43.74 | 6.43 | 44.18 | 5.54 | 43.95 | 6.04 | 0.79 |
| Self-management of emotions | 11.36 | 2.20 | 11.17 | 2.03 | 11.27 | 2.12 | 0.64 |
| Social skills | 11.21 | 2.16 | 11.81 | 1.84 | 11.49 | 2.04 | 0.64 |
| Empathy | 10.63 | 2.34 | 10.98 | 2.01 | 10.79 | 2.20 | 0.71 |
| Utilization of emotions | 10.50 | 2.21 | 10.20 | 2.00 | 10.36 | 2.12 | 0.64 |
| Social problem solving | 48.84 | 11.83 | 47.68 | 11.12 | 48.29 | 11.51 | 0.83 |
| Negative problem orientation | 28.63 | 8.55 | 28.44 | 8.26 | 28.54 | 8.41 | 0.87 |
| Rational problem solving | 14.86 | 5.50 | 13.56 | 5.06 | 14.25 | 5.33 | 0.84 |
| Impulsiveness/carelessness style | 5.34 | 2.02 | 5.68 | 1.88 | 5.50 | 1.96 | 0.66 |
| Hopelessness | 20.66 | 6.15 | 19.85 | 5.73 | 20.28 | 5.96 | 0.88 |

According to Pearson correlation analysis with a Bonferroni-corrected alpha level, there was significant negative correlation between adolescent suicidal ideation and emotional competence ($r = –0.19$, $p < 0.01$). Further analysis showed that there were significant negative correlations between suicidal ideation and three emotional intelligence subscales: self-management of emotions, social skills, utilization of emotions ($r = –0.22$, $r = –0.13$, $r = –0.17$, respectively, $p < 0.01$) (Table 2), providing partial support for Hypothesis 2. However, there was no significant correlation between the empathy subscale and adolescent suicidal ideation. Adolescent suicidal ideation increased when there were lower scores in self-management of emotions, social skills, and utilization of emotions. Since empathy was found to be negatively correlated with suicidal ideation in previous studies[e.g., 37], this variable will be kept in the subsequent regression analysis.

**TABLE 2**
**Correlations between Emotional Competence and Adolescent Suicidal Ideation**

| | Self-Management of Emotions | Social Skills | Empathy | Utilization of Emotions | Total |
|---|---|---|---|---|---|
| Adolescent suicidal ideation | –0.22* | –0.13* | –0.03 | –0.17* | –0.19* |

*$p < 0.01$ (Bonferroni –corrected alpha level).

Standard regression analysis showed that emotional competence was a significant predictor of suicidal ideation ($R^2 = 0.07$). There were no significant differences between the beta coefficients for male and female adolescent suicidal ideation. Increases in all the subscales of emotional competence, except empathy, were related to a significant decrease in adolescent suicidal ideation. All the four subscales were important predictors of male adolescent suicidal ideation, but only self-management of emotions, empathy, and utilization of emotions were important predictors of female adolescent suicidal ideation (Table 3).

**TABLE 3**
**Prediction of Adolescent Suicidal Ideation from Emotional Competence**

| Suicidal Ideation | Emotional Competence | | | | $R^2$ | Adj. $R^2$ |
|---|---|---|---|---|---|---|
| | SME ($\beta$) | SS ($\beta$) | EM ($\beta$) | UE ($\beta$) | | |
| Male | –0.18*** | –0.09*** | 0.10*** | –0.05* | 0.05 | 0.05 |
| Female | –0.22*** | –0.04 | 0.11*** | –0.16*** | 0.09 | 0.09 |
| Total | –0.20*** | –0.07*** | 0.11*** | –0.10*** | 0.07 | 0.07 |

*Note:* $\beta$ = Standardized regression coefficient; SME = self-management of emotions; SS = social skills; EM = empathy; UE = utilization of emotions.

*$p < 0.05$, **$p < 0.01$, ***$p < 0.001$.

Pearson correlation analyses with Bonferroni-corrected alpha level were also performed for social problem solving. Results showed that there was a significant negative correlation between adolescent suicidal ideation and social problem solving ($r = –0.37$, $p < 0.0125$). Further analysis revealed that suicidal ideation was significantly related to the three subscales of social problem-solving, including negative problem orientation, rational problem solving, and impulsiveness/carelessness style ($r = 0.30$, $r = –0.22$, $r = 0.23$, respectively, $p < 0.0125$) (Table 4). Hence, Hypothesis 3 was supported. Adolescent suicidal ideation increased when there was less rational problem solving, but more negative problem orientation and impulsiveness/carelessness style.

**TABLE 4**
**Correlations between Social Problem Solving and Adolescent Suicidal Ideation**

| | Negative Problem Orientation | Rational Problem Solving | Impulsiveness/ Carelessness Style | Total |
|---|---|---|---|---|
| Adolescent suicidal ideation | 0.30* | –0.22* | 0.23* | –0.37* |

*$p < 0.0125$ (Bonferroni –corrected alpha level).

Standard regression analysis showed that social problem solving was a significant predictor of suicidal ideation ($R^2 = 0.14$). All the three subscales were important predictors of suicidal ideation in the total, male, and female sample (Table 5). When the beta coefficients for male and female adolescents were compared[38], the coefficient of negative problem orientation with female adolescent suicidal ideation was significantly higher than that for male adolescent suicidal ideation ($z = 1.98$, $p < 0.05$).

**TABLE 5**
**Prediction of Adolescent Suicidal Ideation from Social Problem Solving**

| Suicidal Ideation | Social Problem Solving | | | $R^2$ | Adj. $R^2$ |
|---|---|---|---|---|---|
| | NPO ($\beta$) | RPS ($\beta$) | ICS ($\beta$) | | |
| Male | –0.21*** | –0.19*** | –0.10*** | 0.12 | 0.12 |
| Female | –0.27*** | –0.19*** | –0.09*** | 0.16 | 0.15 |
| Total | –0.23*** | –0.19*** | –0.10*** | 0.14 | 0.14 |

*Note:* $\beta$ = Standardized regression coefficient; NPO = negative problem orientation; RPS = rational problem solving; ICS = impulsiveness/carelessness style.

*$p$ < 0.05, **$p$ < 0.01, ***$p$ < 0.001.

In the hierarchical regression analysis, when the sociodemographic characteristics were controlled, it was found that emotional competence and social problem solving accounted for an initial 13% of the variance in suicidal ideation. An additional 33% of the variance in suicidal ideation was accounted for by hopelessness. However, emotional competence was no longer a significant predictor when hopelessness was included in the equation. Overall speaking, these results indicate that hopelessness is the most important predictor of suicidal ideation, followed by social problem solving and then emotional competence (Table 6). Hypothesis 4 was thus supported. For the variation of the independent variables in relation to suicidal ideation due to differences in schools, the Wald test of Linear Mixed Model analysis indicated that it was not significant. Hence, there was no random effect of schools.

### DISCUSSION

In response to the limitations in the related literature, there are several unique features of the present study. First, Chinese adolescents were recruited in this study. Second, a large sample based on Secondary 1 to Secondary 4 students was used (n = 5,557). Third, indigenously developed and validated measures of hopelessness, emotional competence, social problem solving, and suicidal ideation were used. Fourth, the relationship and relative importance of the cognitive, emotive, and cognitive-behavioral correlates with adolescent suicidal ideation were examined in this study.

Results showed that parents' marital status was an important predictor of adolescent suicidal ideation. This is in line with previous findings[39]. It is also consistent with the theoretical proposition that nonintact families impair the psychological well-being of adolescent members in the family. In addition, results indicated that fathers' education impacted adolescent suicidal ideation, which corroborates previous findings[34]. A previous study showed that fathers with more education were found to be more confident in parenting, to be more respectful of their children, and to be more warm and involved in child rearing[40]. Since suicidal adolescents perceived their parents to be significantly more critical and less caring, educated parents who were more caring would inevitably have a better relationship with their children, which could have positive impact on the adolescents' psychological well-being and lower their suicidal ideation.

Both correlation and regression analysis showed that a higher level of hopelessness, and a lower level of emotional competence and social problem solving, was associated with a higher level of adolescent suicidal ideation. This observation is consistent with previous literature. Studies have shown that individuals with greater optimism have a reduced risk for suicide ideation in the face of negative life events[2]. Research has also found that adolescents who had inadequate problem-solving skills were at increased risk for suicidal thoughts and behaviors[15]. In addition, there are also findings that show the negative relationship of a person's emotional competence with depression[41].

**TABLE 6**
**Hierarchical Multiple Regression Analysis for the Prediction of Adolescent Suicidal Ideation in the Total Sample**

| | Step 1 ($\beta$) | Step 2 ($\beta$) | Step 3 ($\beta$) |
|---|---|---|---|
| Age | 0.03 | 0.03 | 0.02 |
| Fathers' job status | –0.00 | 0.00 | 0.01 |
| Fathers' education[a] | –0.06** | –0.05** | –0.03* |
| Mothers' job status | –0.01 | 0.00 | 0.01 |
| Mothers' education | 0.01 | 0.00 | 0.02 |
| Parental marital status[b] | 0.08*** | 0.09*** | 0.05*** |
| Family income | –0.01 | 0.01 | 0.02 |
| CSSA recipients | –0.02 | –0.02 | –0.00 |
| Emotional competence | | –0.08*** | –0.00 |
| Social problem solving | | –0.33*** | –0.08*** |
| Hopelessness | | | 0.64*** |
| $F$ | 7.53*** | 67.71*** | 316.76*** |
| $R^2$ | 0.02 | 0.15 | 0.47 |
| Change in $R^2$ | | 0.13 | 0.32 |

*Note:* $\beta$ = Standardized regression coefficient.

[a]Fathers' education (1 = primary education or below, 2 = secondary education, 3 = tertiary education or above); [b]Parents' marital status (1 = intact family; 2 = nonintact family, i.e. single-parent family due to death of a parent, divorce, or separation); CSSA = Comprehensive Social Security Allowance.

*$p < 0.05$, **$p < 0.01$, ***$p < 0.001$.

The present study shows that hopelessness is the most important predictor of adolescent suicidal ideation, followed by social problem solving, and then emotional competence. To explain, Chinese are comparatively more optimistic than their Western counterparts, as reflected in the Chinese sayings that "There must be a way out when the boat approaches the end of the bridge" (chuan dao qiao tou zi ran zhi) or "We have nothing to fear even when the sky falls down" (tian diao xia lai dang bei gai). Conceptually, the sense of hope consists of "certainty about the future" and "future expectation". Previous studies also showed that hopelessness was significantly and inversely related to purpose in life and existential well-being[30]. Hence, adolescents feeling hopeless might be really desperate, and regard suicidal ideation or suicidal behavior as the only alternative available and mistakenly put their hopes on life after death[35]. This explains why hopelessness is the most important predictor of adolescent suicidal ideation. Comparatively speaking, emotional competence is the least important predictor of adolescent suicidal ideation. Since Chinese culture emphasizes emotional inhibition and discouragement of expression of emotions, emotional competence might be regarded as less important by the Chinese adolescents, which in turn contributes to smaller impact on suicidal ideation.

Nevertheless, the present study showed that there were significant negative correlations between adolescent suicidal ideation and three emotional intelligence subscales: self-management of emotions, social skills, and utilization of emotions, with the exception of the empathy subscale. Previous findings also indicated that there was less suicidal ideation among those high in managing others' emotions (i.e., social skills). To elaborate, research showed that adolescents who were good at managing others' emotions tended to have more social support[22]. Besides, people high in social skills helped others to regulate moods in a positive direction. This behavior would lead to more intimate relationships and greater social support, which would help to protect these people from depression and suicidal ideation.

Regression analysis showed that increases in all the subscales of emotional competence, except empathy, were related to a significant decrease in adolescent suicidal ideation. Interestingly, an increase in empathy was related to a significant increase in adolescent suicidal ideation. This could be explained by the fact that an empathetic person, being too sentimental and sensitive to others' emotional expression, might be easily influenced by others' positive or negative emotions. In Chinese culture, some Confucian concepts and principles similar to "empathy" are, for example, "Examine others' views by putting yourself into others' position", "Compare people's hearts with your own". These have a connotation that the person would become less self-centered and more other-centered. The self was suppressed in order to enter others' hearts to understand their feelings and viewpoint. Since the Chinese had strong social orientation, and were attentive and sensitive to others' needs, they were usually too emotionally involved in others' sorrow and misery. Being too emotionally involved might increase personal burden and might not be healthy for individual functioning. Hence, an adolescent might be more prone to developing suicidal ideation when he was empathetic with others' feelings, especially negative emotions.

Of the four subscales, the social skills subscale was not an important predictor of female adolescent suicidal ideation. Traditionally, Chinese females have been described as affectionate, charming, emotional, gentle, kind, and warm. They might be too familiar with complimenting and helping others develop positive emotions. This type of altruistic satisfaction would not be strong enough to protect them against suicidal ideation. Both correlation and regression analysis showed that self-management of emotions was strongly related to and an important predictor of adolescent suicidal ideation. A person's awareness, understanding, and regulation of his/her own emotions were important in decreasing his/her suicidal ideation. This is consistent with previous research on the relationship between emotion regulation, emotion expression, and depression[18]. Although previous literature also revealed that affect dysregulation had been associated with adolescent suicidal behavior, the present study was pioneering in identifying self-management of emotions as an important predictor of adolescent suicidal ideation.

Although there were significant negative correlations between adolescent suicidal ideation and all three subscales of social problem solving, negative problem orientation was the subscale most strongly related to and the most important predictor of adolescent suicidal ideation. This observation was consistent with the previous finding that negative problem orientation was most highly correlated with suicidal risk, followed by an avoidance or impulsive problem-solving style[42]. A Hong Kong study also showed that negative problem orientation had a stronger relationship with measures of family functioning than did impulsiveness/carelessness style[43]. Adolescents with a negative problem orientation tended to view a problem as a significant "threat" to well-being, had self-blame for problems, had low confidence in solving problems successfully, believed that problems could hardly be solved, and were emotionally sensitive to problems. Since they believed that the problems were difficult to solve and there were no other alternatives, they might develop suicidal ideation and think that suicide was the only way out.

Moreover, adolescents who were impulsive might fare even worse as they tended to avoid problems, delayed solving problems for as long as possible, waited for problems to resolve themselves, and attempted to shift responsibility for solving his/her problems to others. Hwang[44] found that besides mobilization of personal resources, seeking help from social resources, and appealing to a supernatural power, Chinese people also tended to adopt the philosophy of doing nothing or avoidance as a coping strategy when facing problems. Furthermore, adolescents with ineffective rational problem-solving skills lacked the abilities and skills to solve the problems. When the adolescents lack rational problem-solving skills and adopt an avoidance strategy, they might be overwhelmed by the problems, thus creating stress and suicidal ideation.

Regression analysis showed that the beta coefficient of negative problem orientation with female adolescent suicidal ideation was significantly higher than that for male adolescent suicidal ideation. Since females had more negative problem orientations, greater avoidance tendencies, and less ability to generate alternative solutions, they tended to turn to suicidal ideation more easily, while males might resort to alternative ways of coping even though they had a negative problem orientation. There is a need to further investigate why negative problem orientation is more significantly related to female suicidal ideation than male suicidal ideation.

From the above discussion, it can be shown that the results are consistent with the cognitive, emotive, and cognitive-behavioral theories that postulate hopelessness, emotional competence, and social problem solving have impacts on suicidal ideation. Although hopelessness is the most important predictor of suicidal ideation, social problem solving and emotional competence should also be considered as key building blocks in theoretical models for adolescent suicidal ideation. This adds evidence to the cognitive, emotive, and cognitive-behavioral models in explaining adolescent suicidal ideation in the Chinese context. However, emotional competence has the least impact on adolescent suicidal ideation.

Practically speaking, it is recommended to use an integrative cognitive-emotive-behavioral approach to reduce adolescent suicidal ideation. Since the cognitive correlate hopelessness is positively related to adolescent suicidal ideation, it is important to cultivate the sense of hope in adolescents. A study has shown that increasing one’s positive future expectations, and identifying and pursuing one’s life goals and plans, could reduce suicide risk[45]. Workshops and programs can be conducted in order to help adolescents build up hope and future expectations, develop positive attitudes to exploring possible options in life, and recognize their own capabilities in goal achievement. Some adventure-based counseling, i.e., implementation and debriefing of adventure activities to enhance self-understanding, coping with adversity, and interpersonal cooperation, can be introduced so that adolescents can learn to persevere in the face of hardship.

In addition, it is important to enhance emotional competence, the emotive correlate, among adolescents. Buckley et al.[46] reported that mastery of the skills of emotional competence was contributive to positive youth development. Specifically, groups and workshops could be organized for adolescents in order for them to understand and regulate their own emotions and manage others’ emotions, as well as make effective use of emotions. Although there are some local programs, e.g., “Positive Adolescent Training through Holistic Social Programs” (P.A.T.H.S.)[47,48,49,50], to develop students’ awareness of their emotions and proper emotional expression, there is a lack of emphasis on social skills and the utilization of emotions, which are significant predictors of adolescent suicidal ideation, in these programs. Since the Chinese adolescents were socialized to suppress their emotions and an “ideal” person was characterized by self-constraint and control of emotions in Chinese societies, practitioners should also take note of this unique problem of Chinese people regarding emotional expression and the possible cultural barrier in conducting such workshops and programs.

Since the cognitive-behavioral component, social problem solving, is negatively related to suicidal ideation, it is important to enhance rational problem solving skills and decrease negative problem orientation and impulsiveness/carelessness style among adolescents. A local study showed that cognitive-behavioral group intervention, introducing rational thinking and coping skills, was effective in helping persons at risk of developing mental health problems in Hong Kong[51]. The P.A.T.H.S.[52,53,54,55], which included problem solving and resilience training, was found to be effective in reducing the negative behaviors of secondary school students. However, an integrative intervention approach comprising all the cognitive, emotive, and behavioral elements is recommended.

Because adolescent social problem-solving abilities were not strongly encouraged in Chinese families, it might be difficult for adolescents to adapt to learning such skills. Practitioners should try to change the adolescents from being negative and avoiding problems to active coping attitudes when facing problems. Workshops and programs could be held to teach the adolescents rational problem-solving skills, such as problem definition and formulation, generation of alternative solutions, decision making, solution implementation and verification, as well as to cultivate a positive problem orientation and to introduce active coping strategies.

It is noteworthy that there are several limitations of the present study. First, schools have been approached through agencies providing school social work service and the resulting samples thus represent convenience samples. In particular, generalizing the present findings to the broader secondary school population should proceed with caution. Second, as self-report questionnaires were used, the information collected was based on the adolescent informants only. If resource permits, the use of multiple informants would give a clearer picture of the problem area. Third, the present research predominately used quantitative methodology. One disadvantage of this methodology is that it cannot explore the subjective experiences of adolescents. Another drawback is that it is less process oriented and difficult to apply the generalized data to individual cases. Fourth, besides the correlates utilized in this study, other cognitive, emotive, and cognitive-behavioral indicators should also be used in the future. Fifth, the use of a cross-sectional design means that it is not possible to claim a causal relationship between the antecedents and adolescent suicidal ideation. There may, in fact, be a bidirectional relationship between the variables or a third variable may be present. Hence, a longitudinal research design can be used in future studies. Despite these limitations, the present study highlights the importance of cognitive, emotive, and cognitive-behavioral factors in adolescent suicidal ideation.
